# “Imagine You Have ALS”: Death Education to Prepare for Advance Treatment Directives

**DOI:** 10.3390/bs11010006

**Published:** 2021-01-06

**Authors:** Ines Testoni, Lorenza Palazzo, Nicoletta Calamarà, Gabriella Rossi, Michael Alexander Wieser

**Affiliations:** 1 Department of Philosophy, Sociology, Pedagogy and Applied Psychology (FISPPA), University of Padova, 35131 Padova, Italy; lorenza.palazzo@unipd.it (L.P.); nicoletta.calamara@gmail.com (N.C.); 2 Emili Sagol Creative Arts Therapies Research Center, University of Haifa, 3498838 Haifa, Israel; 3 Unione Italiana Lotta alla Distrofia Muscolare (UILDM)—Milano Section, 20124 Milan, Italy; rossi.gabriella@yahoo.it; 4 Department of Psychology, University of Klagenfurt, 9020 Klagenfurt am Wörthersee, Austria; Michael.Wieser@aau.at

**Keywords:** death education, community, advance treatment directives, amyotrophic lateral sclerosis

## Abstract

The study presents the results of qualitative research carried out within a death education project dedicated to advance treatment directives (ATDs) in which it was proposed to participants to empathize with people who had received a diagnosis of Amyotrophic Lateral Sclerosis (ALS). The study involved 104 people who discussed and reflected on issues related to the knowledge of having to die, palliative care and ATDs, investigating what choices they would have made if they had received such a diagnosis. Finally, they were asked to write a paper describing their impressions and hypothetical choices. Qualitative analysis has elucidated among fundamental themes. Four thematic areas emerged from the data analysis: (1) ATDs and the family; (2) the importance of reducing pain and suffering; (3) emotions and considerations regarding death, illness and spirituality; and (4) opinions on the DeEd course. It has emerged that some people are unfamiliar with palliative care or the right to self-determination and that addressing these issues helps manage the thought of the future with less terror. The experience of death education has therefore proven to be very positive in dealing with complex and often censored issues, allowing thinking about death in a less distressing way.

## 1. Introduction

Italy is a country where the right to advance treatment directives (ATDs) is recent because of ideological contrasts that have kept public opinion strongly divided for many years, despite the fact that the right to health and self-determination of the patient was originally admitted by the Constitution in art. 32 paragraphs 1 and 2 and has been reaffirmed by the Supreme Court (judgement 21/04/1992); by the Oviedo Convention of 1997, ratified and implemented by Law 28/3/2001 n. 145; by the Charter of Fundamental Rights of the European Community and the Code of Medical Ethics of 18 May 2014; and by the Constitutional Court judgement n. 438/2008. On 22 December 2017, ATDs were approved by Law n.219 ‘Rules on informed consent and advance treatment directives’ (G.U. n. 12 of 16/01/2018) inherent to informed consent, shared care planning and ATDs. ATDs (Article 4) represent the expression of the unilateral initiative of a person, while the shared planning of care (Article 5) concerns a process that originates with and involves the doctor/patient relationship. Despite the importance of all this, ATDs are still infrequently used, and people are poorly prepared to handle them because of lack of information and shared elaboration of their representations.

Among the diseases that require the most preparation at this time, we can include amyotrophic lateral sclerosis (ALS). ALS is a progressive neurodegenerative disease with an inauspicious prognosis that affects the upper and lower motor neurons of the cerebral cortex, trunk and spinal cord [1,2]. ALS is a rare disease that annually affects 1–3 people every 1,000,000 and decreases rapidly after the age of 80. It is associated with a survival of about 3–5 years after onset, although there are cases where survival can reach 10 years [3,4,5]. There are currently no healing methods but only palliative treatments, so in the management of the disease it is of paramount importance to respect the self-determination of the patient, who must be fully and transparently informed not only about the diagnosis but also about possible evolutions of the disease, so that he or she can make informed and responsible choices about the treatments to be received or refused [6]. A proper doctor–patient–family relationship must be established to achieve the best results and not to let the patient and their family fall into despair [7]. In the case of ALS, since it can evolve to the point of preventing the patient from expressing his or her will due to the absolute loss of voluntary movements and/or cognitive impairment, the sick person must be able to express his or her will at an earlier stage, and shared treatment planning is the best tool for this purpose. This is an optional choice, but not doing so could expose the patient to the risk of leaving their wishes unexpressed [8]. During the media conflicts that characterized the period before the approval of Law 219/17, in particular after Piergiorgio Welby’s letter to the President of the Republic, Giorgio Napolitano, in 2006 in which he asked to be able to suspend the invasive treatments that kept him alive, neurodegenerative and muscular pathologies that lead to progressive paralysis have become increasingly known, even among ordinary people. ALS can therefore be considered a pathology that can be taken as an example for understanding the importance of ATDs in courses aimed at raising social consciousness on these issues.

The term death education (DeEd) refers to a set of activities aimed at facilitating the understanding and acceptance of death within a postmodern and secularized cultural context in which this theme is censored and considered taboo, and death is confined to the hospital environment [9,10,11]. These cultural transformations have produced the need to resume dialogue and reflect on death and related concerns since the avoidance of this topic, especially in Western societies, has become very common [12,13,14]. DeEd responds to this need, as well as the need to promote reflections on existential issues, and it leads to the management of emotional difficulties related to loss, subsequently reaching a greater sense of control over the representations of death and the anxiety related to it [14,15,16]. DeEd can be achieved at different levels of prevention: primary, secondary and tertiary [11,13,17,18]. DeEd as primary prevention, on which the study reported in this article is focused, is implemented in situations where there is no recent experience of death but the topic is addressed in a preventive way, focusing on sharing one’s emotional experiences and building an appropriate and shared language [19,20]. Experiences of DeEd as primary prevention have shown that it does not produce negative effects but allows better management of related anxiety, decreases alexithymia, increases empathic understanding of others and approaches them by learning to understand their viewpoints and emotional experiences and to re-evaluate aspects of one’s life [13,19,20,21]. Starting from different multidisciplinary contexts, DeEd can be carried out through moments of “formal” theoretical lessons, combined with “informal” lessons that may include imaginative techniques and art therapy experiences such as photovoice, phototherapy, and psychodrama [13,18,19,22,23].

## 2. Materials and Methods

### 2.1. Aim of the Study

First of all, the researchers wanted to offer a space to reflect on ATDs by reflecting on death without creating anxiety. The goal was to allow participants to activate the processes of identification to deal with their representations and express them. Thorugh the analysis of these accounts, the study aimed to explore what choices people might hypothetically make if they were diagnosed with ALS and how they would behave with respect to communicating their treatment choices to their relatives and children. The researchers also wanted to consider the effect gained from this experience and to know participants’ opinions with respect to the DeEd course.

### 2.2. Procedures

The study was carried out during two courses of community DeEd with adults motivated to participate in the initiative; it involved healthy people and was aimed at reflecting on the meaning of serious illness, end-of-life and death in order to understand the usefulness of ADTs, after the approval in Italy of the law that made them possible. The Italian situation is in fact very particular because, before the approval of the law, the conflict between those who wanted to limit the self-determination of patients and those who instead wanted to enhance it was very heated and particularly violent at both political and media level. The intervention was carried out in two Italian cities by a psychologist expert in DeEd and lasted 4 h. Initially, there was a formal part on the history and function of DeEd, on the relationship between life, illness, medical intervention and death, and on the legal aspects related to end-of-life decisions. In addition, ALS as a serious incurable disease was presented at an illustrative level, because it is perhaps the most commonly known neurological disease. In the second informal part the experience of Piergiorgio Welby was used as an example, through the screening of a movie about his story. In the last hour the participants were asked to put themselves in the shoes of an ALS patient who has just received the diagnosis and to draw up their own ADTs, expressing their wishes about the treatments to be received or refused, and what they would like their relatives and children to know about their disease and prognosis.

The participants were informed of the objectives and procedures of the research, and the confidentiality of their answers was guaranteed.

The study followed the APA Ethical Principles of Psychologists and Code of Conduct and the principles of the Declaration of Helsinki. It received research ethics approval from the Health Sciences and Science Research Ethics Committee of the University of Padova (reference: F7185F35F0C57FF30D9ED3DE246933A5). Participants provided written informed consent before participating in the study.

### 2.3. Participants and Method

The project involved 104 people, 79 females and 25 males aged between 22 and 73 years (M = 50; SD = 12); all had a Catholic education but not all were practicing Catholics, and half of them were atheist or agnostic. With respect to employment, the participants included 25 health professionals, 14 freelancers, 13 employees, 9 retirees, 25 teachers/educators, 3 members of religious orders, 2 students, 2 unemployed people and 2 returning other occupations; 9 people did not respond.

As indicated above, the Italian situation is characterized by high level of conflict on these topics and there is still a significant lack of studies concerning people’s ATDs competences and their psychological representations. Therefore, the preferred methodology to better explore this area was the qualitative research approach that allows the researcher to bring out people’s points of view, embracing their complexity [24]. In particular, the strategy of the Thematic Analysis (TA), which developed from the Grounded Theory, was chosen because TA is widely used in the psychological studies inherent to health and wellbeing. This methodology allows researchers to identify and examine the sources in terms of the concepts or main themes within a text. Specifically, the TA approach used was the semantic one, that is, themes were identified within the explicit meanings reported by the participants and the researcher did not look for anything beyond and tried to recognize the perspective of participants [25]. The analysis process was divided into several stages: preparatory organisation, code generation, data coding, understanding of the elements that emerged, searching for alternative explanations and writing the results. The texts obtained from the ATDs drafted by the participants are digitized and, together with the answers collected on the online platform, have been processed using the qualitative analysis software Atlas.ti [26,27]. After coding all the texts, similar codes were merged, from which four main thematic areas emerged. All steps were performed by two researchers who constantly compared their notes. A third researcher then evaluated all interpretations and the three researchers together chose the clearest and most informative ones.

## 3. Results

Four thematic areas emerged from the data analysis: (1) ATDs and the family; (2) the importance of reducing pain and suffering; (3) emotions and considerations regarding death, illness and spirituality; and (4) opinions on the DeEd course. The quotations reported have been modified and adapted to preserve the anonymity of the participants. For the same purpose, fictional names were used in reporting the answers.

### 3.1. First Thematic Area: Advance Treatment Directives and the Family

Most participants said that they would openly communicate about their illness with their partner and any children. Regarding the moment in which to make such a communication, some would do it immediately, while others would tell their partner first and then their children because of the need to clarify the situation for themselves first. On how to communicate the diagnosis, some said that they would use the support of trusted people and/or professionals to explain to their children what was happening, as in the case of Marta:

*‘My will regarding my children is the following: I want them to be informed about the disease and its course by people who are close to them, in turn well educated on what to say. If it is still possible for me, I will participate myself in the conversation with them, in solitude. If not, I want them to be informed by people they know and love’.*

Similarly, Maria said:

*‘I would like my children to know about the disease, but I am not sure I can find the right words to explain it to them, which is why I would prefer my psychotherapist friend to do it. After they heard about it, I would like to discuss it with them in a quiet and conscious way’.*

From the texts the importance emerged of adapting the communication of the diagnosis and their wishes according to the age of the children to inform them in a sensitive way, maintaining clarity and honesty, as Luca reported:

*‘I would try to explain to my children, in a simple way and through the use of metaphors, the situation and what will have to be faced. I would try not to scare them, I would try to make them understand that death is a passage and a transformation’.*

All participants stated that they wanted to share their decisions regarding treatments with their families and significant people (partners, relatives, friends and trusted people). As provided for by Law 219/17, the author of the ATD can appoint a trustee, who will have the task of acting on behalf of his principal from the moment in which the latter has lost all ability to understand and want. The participants expressed their decisions indicating several persons among the possible trustees, including a partner, son, spiritual father, friend, brother or sister, sometimes even a notary. All participants expressed their willingness to make full use of Law 219/17, being able to suspend any type of treatment when their mental capacity and lucidity fails, but before that moment they have the opportunity to change their decisions based on the course of the disease, meetings with doctors and discussions with family members. Some participants, although expressing willingness to express their ATD to their loved ones, simultaneously expressed the fear that their decisions could become an emotional burden for others. This concern was also expressed by the participants concerning the choice of wanting to benefit from all possible care to maintain their autonomy and not weigh and overburden their loved ones, as in the case of Mara:

*‘I want to be treated for my disease, but only as I am able to eat alone and wash myself. When I am no longer self-sufficient and can no longer move, I do not want to be subjected to any kind of treatment or therapeutic relentlessness, and I do not want to be kept alive by any machine’.*

Similarly, Claudia said:

*‘Besides not bearing the idea of being a burden to my loved ones, I don’t want to be in such a condition that I can’t play an active role in my life. I want to breathe independently, feed myself independently, communicate without the help of external means’.*

### 3.2. Second Thematic Area: The Importance of Reducing Pain and Suffering

Most participants said they would like to take advantage of all those treatments that can relieve pain and suffering and help them in the actions that they are no longer able to perform, such as non-invasive devices, and to reject the more invasive ones such as percutaneous endoscopic gastrostomy [PEG], tracheostomy and therapeutic fury in general, like Roberta:

*‘I would like to live on my own strength and, at the moment in which they should fail, I would like not to be attached to respiratory machines. I wish I could be sedated to avoid atrocious suffering’.*

Others, however, would accept invasive treatment to spend more time with loved ones, as in the case of Nicola:

*‘It would be difficult to accept to be intubated and to resort to PEG, but I believe that I would not have the courage and firmness to give it up, I trust in the possibility to get used to it and have, consequently, more time to spend in the company of my family’.*

Almost all participants expressed the fear of suffering and the fear of dying from suffocation and, for this reason, they expressed the request to receive intermittent sedation, in some cases even deep sedation, also connected to the desire not to want access to treatments considered invasive to protect their dignity, as in the case of Mario:

*‘I would accept the therapies and treatments that allow me to live as long as life is dignified. I ask not to be subjected to invasive interventions, but to be subjected to deep sedation, when my condition is critical, to ensure my dignified survival’.*

Finally, the last significant need participants expressed was the wish to be cared for at home, as in Eleonora’s case:

*‘If possible I ask to be cared for at home as long as possible with palliative care. The ideal would be to be able to spend my last days of life at home, taking advantage of nursing care. If possible, I ask to be cared for at home as long as possible with palliative care’.*

### 3.3. Third Thematic Area: Emotions and Considerations Regarding Death, Illness and Spirituality

The third thematic area allowed us to highlight the prevailing emotions that participants think they would feel after receiving the diagnosis of ALS, during the course of the disease and concerning death. Among those that emerged more frequently were fear, dismay, repudiation, rejection, fear of not bearing the stress of the disease, and disbelief. Some participants also used metaphors to define ALS, for example, as a “nightmare”, “ordeal” and “terror”. Participants described the emotions related to the fear of dying, stating that they would not be able to handle the weight of the idea of no longer existing and would also feel a marked concern for family members who remained alive, feeling pain at the mere idea of having to abandon them, as in the case of Agostino:

*‘Needless to say, I am afraid! I strongly fear that I am unable to psychologically manage the burden of the idea of not existing, and the fear of leaving my children and my family without me’.*

The concern for the loved ones who will have to face the loss has led many participants to express the desire to be able to entrust their children to people who are prepared and trustworthy when they are gone, as in Sara’s case:

*‘I ask for psychological assistance for my son so that he can understand my choice without living my absence with pain and regret, but with naturalness, perhaps with the help of specialised people’.*

Other participants said that they would hide their emotions and their concern from their children, so as not to make them sad and to leave them with beautiful memories; many of these participants also said that they would never want to die in front of their children. In particular, Rosanna described this clearly:

*‘If I wasn’t able to breathe on my own, I’d require deep sedation. In this case, I think it is important that I, together with my husband, explain to our children the reasons why I am leaving this life for what is to come. I would like to do so by leaving strong in them the conviction that I love them very much, that I will always be close to them, and that we will meet again in the next life. I would like to leave them a serene memory of their mother and transmit to them the desire to live their lives intensely and the confidence in another. I would like to give them a last goodbye, but that they were not present at the moment of the sedation, that is, at the moment of death. I think it is essential that there is a moment of farewell that allows them to say everything they consider important so that they do not have the remorse of not having said something’.*

Others expressed the wish not to be left alone at the moment of death, requiring the presence of the most intimate people, including children, during their passage, as in the case of Claudia:

*‘I would like them and my husband to accompany me on the path of illness until death, hoping that it will not be too long and tormented for them. I would like, if the situation would allow it and without making my children feel too uncomfortable, that they would stay in the room with me at the moment of my death. What is most important to me is that they are surrounded by people who love them and can understand whether it is preferable to send them away’.*

The awareness that they may be suffering from a disease with an inauspicious prognosis has allowed the participants to reflect on the disease, not necessarily perceiving it as a threat but as a lesson to themselves and their loved ones, especially when communicated sincerely because the truth can facilitate the process of acceptance. The evidence of mortality has allowed participants to reflect on the time lived and wasted, especially the need to value moments with loved ones. Many have written that they would try to make sense of what was happening, preparing themselves and their loved ones for the inevitability of death, even with the support of specialists, resolving unfinished business, leaving lessons for those who remained alive. From these thoughts, it also emerged that some participants see illness and death as a life lesson and an experience from which something can be learned, as in Piera’s case:

*‘I want my family to know how things really are and to have the possibility to be close to me and to approach the mystery of death, with the help of an intelligent spiritual guide. May my death be a moment of transformation and growth for more conscious lives’.*

Thinking about the illness has provoked reflections on the meaning of death, like those of Bruno:

*‘But let’s all think about it for a moment, death is not so frightening, it’s not so serious, it’s not even so definitive. Everything we had together remains, even the next period together will be beautiful even if painful; let’s enjoy everything together, let’s not miss anything, that the spectre of death makes every moment wonderful and precious together. For when it will no longer make sense, let me go, let life and death take their course, let me go into a new dimension where maybe we will meet again or maybe not, but what we had together will not be erased, it will remain ours’.*

As for religiosity and spirituality, about half of the participants claimed to be atheists or agnostics, and half were religious or relied on their spirituality. Believers stated that they would face the disease by finding shelter in their faith, as in the case of Jessica:

*‘As a Catholic and deeply believing as I am, I would like to have as much spiritual accompaniment as I can, doing whatever it takes to get the best out of the other life. I would like to do this with my family, with my spiritual father if he will be there, and with those who have shared my journey loving me’.*

Some also mentioned the importance of their “spiritual will”, considered a permanent trace of themselves, a memory, or a word of comfort to their loved ones that can remain in time, even after they have died, as Francesca said:

*‘I will, however, leave a letter for each of my family members, so that in time they can accept and understand my choices, my way of life’.*

### 3.4. Fourth Thematic Area: Opinions on the Death Education Course

The experience of reflection offered by the DeEd course was judged positively by all participants, such as Federica:

*‘It was an important experience of reflection on death. It is a subject on which one never dwells’.*

Similarly, Carolina said:

*‘It was interesting and unusual, we rarely talk about death and reflect on these issues. I was struck by the fact that we talked about illness and living wills, which are absolutely unusual topics. The idea that most impressed me is the close link between life and death’.*

Stefano instead highlighted the emotional aspects:

*‘I lived the proposed experience with interest and great emotional involvement. I felt shaken internally because I seem to have become even more aware of my limits, but also of my resources. I became aware that I am small and alone, but also that I need others, especially spiritual friends’.*

Even those who felt more reluctant to address the subject expressed positive opinions, like Tiziana:

*‘I lived this experience of DeEd with great interest. Even if I keep my distance from this topic, I have reflected on the different topics trying to understand what I agree on among the many things that have been said and done’.*

Similarly, Daniela said:

*‘At first I lived this experience with fear, then I realised that I had been thinking about these problems for a long time, but without having ever made them explicit to anyone. It is important to be able to confront these problems. I think I have made a decision about how I will manage the end of my life, but I do not know if my thoughts will remain the same if I have a negative experience’.*

For almost everyone, the experience was an opportunity to gain serenity as, for example, Andrea said:

*‘It was an educational experience that allowed me to reflect. It was all very intense because we tried to remove the censorship that characterises Western culture on the themes of death. The experience of DeEd was partly a revelation, partly a confirmation of my personal opinions, but I have never spoken about them before. All this has given me serenity and clarity’.*

For some participants, the serenity came from understanding what palliative care consists of and what ATDs consist of, as, for example, what happened to Anna Paola:

*‘Thanks to this meeting of death education, I learned that there are palliative cares that can soothe my suffering. I did not know that. Knowing this thing has given me serenity and also increased my sense of freedom because now I know that I can stop the treatment and I can resort to terminal sedation’.*

Finally, it was stressed by almost everyone how reflection on death teaches us to value life, as Lucia said:

*‘It was definitely a highly formative experience. It confirmed my beliefs about death and the meaning of life. Living life with the awareness of a finite and limited time gives a greater awareness and commitment to realise our projects and experiences. I think it is important to give the same awareness to the children, too’.*

## 4. Discussion

The study found that DeEd is a good instrument for bringing people closer to the topics of illness, death, and ATDs. Four dominant themes emerged from the thematic analysis: the way of managing ATDs within the family and the relationship with children; the importance of reducing pain and suffering and the negative emotions with respect to death and disease, but also the importance of spirituality for relieving this negativity. Finally, the opinions on the DeEd experience produced very useful results and were judged positively even though the experience was challenging. In fact, the course proved to be a beneficial opportunity to shed light on some issues surrounding dying that are still little known not only by the general Italian population but also by health professionals [28].

More specifically, the experience of DeEd concretized through an imaginative experience of identification with ALS patients allowed participants to imagine the possible emotional reactions that characterize the experience of those who suffer from severe illnesses. The choice of using ALS as a prototypical sickness was helpful because this disease is very well known. This allowed to facilitate the phase of identification with the patients that the educational experience proposed. Although it is unequivocal that there is a huge difference between being ill and imagining to be ill, this experience offered the possibility to approach the theme of death without experiencing excessive anxiety [19].

The participants showed that they could imagine the experiences of the strong fear, anger, helplessness, despair, worry, desire to keep their autonomy intact, and fear of weighing on their loved ones that characterize such an experience, as already described in the literature [29]. In particular, the fear of losing one’s autonomy and becoming a burden on one’s family is a trait of particular relevance, to which the study offered specific attention [30,31,32,33,34] because the demand for assisted suicide and euthanasia is often inscribed in this scenario [35,36,37,38]. Among the factors that participants consider fundamental to avoiding desperation are freedom of choice concerning the end of life and the use of invasive devices. Almost all participants declared that they would refuse any situations constricting them to live without their autonomy and dignity but also stated that they might change their opinions in the future based on their experience with the illness. This is what happens in reality. In fact, people often change their minds as their illness progresses. However, those who do not know that they can modify their preferences and write as many ATDs as they wish will find it more difficult to accept this practice. In fact, the literature stresses how important it is to emphasize to patients that the ATD can be revised at any time by asking for help from any healthcare professional [39].

Among the factors that the participants considered as possible strengths in managing the suffering and stress caused by sickness was the factor of religiosity/spirituality, because this dimension gives meaning to negative experiences and conditions, helping to manage the terror of death. It is also considered useful to strengthen family members in the accompaniment and care of their sick loved ones. This is already confirmed in the ALS literature, which describes how these patients are facilitated in giving meaning to the disease by leveraging spirituality and/or religious beliefs and how this is related to greater well-being, less depression and less anxiety [40,41,42,43], reducing requests for euthanasia and assisted suicide [44]. The coherence between the representations of the participants and what is conspicuously highlighted by the literature is in line with the Terror Management Theory according to which distal, i.e., cultural, defences help patients to think about existence beyond death and better manage death anxiety [45,46,47,48,49,50].

The ALS imagery of our participants highlights the need to enhance meaningful relationships within and outside the family, including the support of trusted and/or specialized people, such as doctors and psychologists. A need highlighted is communication, which has already been considered in the literature as a problematic dimension that requires special care [51,52,53,54,55,56,57]. Within this area, the theme of how to communicate the diagnosis of an illness with an unfortunate outcome to children has been particularly noteworthy. Almost everyone has expressed the importance of being able to involve their children in the experience of pain and treatment, but up to a certain point, that is up to the moment of the shared goodbyes that must take place before death and terminal sedation. It is important to help patients talk to and confront their children openly to safeguard the authenticity of their relationship and prevent third-party interference. The importance of involving children in this process has not been particularly studied in the literature and seems to emerge as a significant issue to be considered for the activation of resilient dynamics within the family and in children [58,59].

Great emphasis has been placed on the figure of the trustee. For all participants, it was vital that their desires were respected [60] as well as having the possibility of changing the ATD at any time, as already highlighted by the literature [61,62,63]. This idea was stressed considering the difference between imagining being sick and being sick. In the participants’ opinions, this difference requires that someone can protect the will of the sick through clear and transparent communication with the family members and the medical team [64,65,66]. In particular, it was considered crucial to respect the decisions regarding therapies that require more invasive interventions, while palliative care was highly valued. It was underlined that it would be essential to develop spaces for reflection that allow people to better understand how these steps can be managed by sharing care plans among caregivers, patients and health services [67,68,69]. This instance is linked, in the imaginations of our participants and in the realities of actual patients, to the idea of dignity linked to the maintenance of positive social relationships [70,71,72]. Along these lines, some participants mostly underlined the desire to die serenely, possibly without being alone during passage, surrounded by loved ones in a planned and shared way, thanks to deep sedation. This data confirms what has already been described in the literature, which shows how the possibility of deciding how to die helps to manage death anxiety [73,74]. The expressed need to face the hypothetical disease with loved ones, avoiding being left alone, also confirms that significant emotional relationships are protective factors for the quality of life of ALS patients, able to increase levels of emotional and physical well-being [75,76,77,78,79,80].

## 5. Conclusions

This study confirms how community death education enables people to get to the heart of what it means to face a serious illness such as ALS and then manage palliative care with family members. DeEd can get ordinary people to think of death and dying before they are diagnosed with a serious illness. DeEd can help individuals to reflect on issues that they do not normally give importance and that need to be pondered instead. In fact, holding these issues in due consideration on the one hand can be useful for managing way the possibility of the disease in a more resilient, and on the other hand for developing more feelings of solidarity and empathy towards patients and their families. This project found that people are not sufficiently knowledgeable about either palliative care or the law governing ATDs. Even these people have considered the experience of death education useful for becoming aware of this possibility, and this has increased their serenity. This confirms that it is possible to talk about death and the most dramatic situations of illness without creating anxiety, but reducing it. What reduces anxiety is the possibility of talking about these issues and becoming aware of how culture and society manage them, offering important answers that are usually hidden from everyday life. This study confirms the relevance of religious and spiritual discourse, which is considered particularly valuable when addressing these issues.

## 6. Limitations and Future Perspectives

The most important limitation of this study is that the results cannot be generalized, not only because the research design is qualitative, but also because it is inevitable that people’s intentions change, especially in the transition from being healthy (like the participants in this experience) to being sick. Future explorations may investigate whether sick people adopt the same ways of dealing with this issue. A further limitation concerns the impossibility of generalizing results with respect to the possibility of applying the same methodology to other disorders, e.g., neuromuscular diseases. In fact, ALS is well known for the attention that the media dedicate to it, while other neuromuscular diseases are less well-known and therefore the description could be more accurate and time consuming. Certainly, it may be useful for future investigations to develop DeEd courses with other examples and survey their effects. Another limit is that the competence of the participants with respect to ATDs and shared care planning, prior to the death education experience, was not verified. Future studies could be carried out with targeted courses that would allow clarity on these aspects. This could permit us to analyse the representations of the differences between deep sedation and euthanasia to recognize any possible confusion between them. In fact, in this study, participants did not quote euthanasia and assisted suicide, despite often citing deep sedation as a positive solution that permits sick persons to die serenely. In order to better develop the analysis of the Italian cultural context, since deep sedation is not substitutive of euthanasia and assisted suicide, it could be very meaningful for further studies to consider this issue among two groups of participants, religious people and atheists, comparing them.

## Data Availability

The datasets used and/or analysed during the current study are available from the corresponding author on reasonable request.
